# Advancing Diagnostic Equity Through Clinician Engagement, Community Partnerships, and Connected Care

**DOI:** 10.1007/s11606-022-07966-8

**Published:** 2023-01-05

**Authors:** Traber D. Giardina, LeChauncy D. Woodard, Hardeep Singh

**Affiliations:** 1 Houston Center for Innovation in Quality, Effectiveness & Safety (IQuESt) (152), Michael E. DeBakey Veterans Affairs Medical Center (MEDVAMC), Houston, TX USA; 2 Department of Medicine, Baylor College of Medicine, Houston, TX USA; 3 Department of Health Systems and Population Health Science, Tilman J. Fertitta Family College of Medicine, University of Houston and Humana Integrated Health System Sciences Institute, Houston, TX USA

While the COVID 19 pandemic exposed longstanding race- and ethnicity-related healthcare inequity in the USA, equity issues specifically related to the diagnostic process warrant additional exploration. For example, Black, Hispanic, and Asian patients experience significantly higher rates of infection, hospitalization, and death from COVID-19, yet are less likely to be tested for coronavirus.^1^ Similar disparities have been documented with cardiac testing and procedures, mental health diagnoses, appendicitis diagnosis, and receipt of diagnostic imaging in the ED.^2^ Diagnosis-related inequity is particularly challenging to address especially given that diagnostic errors have been inadequately targeted by systems-based solutions.

We recommend the following definition of *diagnostic inequity*: the presence of preventable unwarranted variations in diagnostic processes among population groups that are socially, economically, demographically, or geographically disadvantaged. About 1 in 20 US adults experience a diagnostic error in the outpatient setting annually,^3^ but this likely underestimates frequencies for marginalized patients, who face additional biases, discrimination, and structural factors.^4^ Diagnostic error measurement is limited by lack of adequate data sources and rigorous and standardized methods, and precise epidemiologic data are not available. But, for marginalized patients, prevalence and factors contributing to diagnostic errors are even further underexplored—particularly where multiple social identities intersect (e.g., misogynoir). Mitigation strategies are not well developed but are urgently needed. Given the barriers marginalized patients face, several existing strategies could be leveraged that include engagement of key stakeholders as partners and leveraging digital healthcare solutions to identify and address unjust norms. In this paper, we propose a three-pronged strategy focused on advocacy and partnerships to begin to address diagnostic inequity: clinician engagement, community partnerships, and connected care.

## ENGAGING CLINICIANS 

Engaging clinicians as advocates is essential to dismantling diagnostic inequity. Clinicians are in a unique position because they not only experience the structural constraints of the healthcare system but also personally witness the many vulnerabilities that patients experience. Furthermore, they hold significant power in the diagnostic process and their biases can perpetuate disparities if not adequately addressed. Unconscious biases can be present even in those whose professional ethics include an obligation to patient safety and equity.

Implicit associations of positive and negative attributes with particular patient populations can influence clinicians’ judgments, leading to harmful biases in practice. In addition to affecting judgments, biases impact behavior toward others (e.g., reduced eye contact or physical interactions). While there is debate on the extent that these biases impact clinical decision-making, clinician bias is associated with poorer patient-clinician communication. The notion that clinicians contribute to inequity, particularly through bias, may not align with clinicians’ existing beliefs about their role in healthcare disparities. Despite an increasing commitment to reducing bias through training and curriculum, there is limited evidence about how to best communicate with clinicians about disparities in their work.

The educational concept of critical consciousness, which focuses on awareness of social, cultural, and historical dynamics that contribute to inequities^5^, has potential to encourage an orientation toward social justice. It requires clinicians to examine policies and actions that perpetuate inequity among their patients within their clinical practice and healthcare systems. Clinicians may be motivated to address their biases through self-reflection activities (e.g., examining rates of how often essential testing is offered) and connecting with values that are important to reducing bias (e.g., all patients deserve quality care). For diagnostic safety, this means developing awareness of inequities within the diagnostic process, and positioning clinicians as crucial advocates for reducing disparities in the diagnostic process.

Segmented data (e.g., subsets of data) may be a method to sensitize clinicians to biases within the diagnostic process and create opportunities for augmenting critical consciousness. This approach would require modifying current systems used to identify patient safety events (e.g., incident reporting, electronic algorithms). These systems do not adequately identify events in vulnerable patient populations, even though vulnerable patients are at higher risk of patient safety events.^6^ In practice, this would mean the use of personalized, segmented data on diagnostic safety events broken down by patient factors (e.g., race/ethnicity, gender, insurance, comorbidities) would uncover existing biases. Framing narratives effectively while evaluating segmented data and leveraging diverse clinician peer-to-peer learning and interactions may help engage clinicians in critical consciousness. When used as a problem-solving approach, critical consciousness may activate clinicians to advocate for systemic change within their organizations by making them aware of their own biases and the impact of their actions and behaviors on their patients and colleagues.

## COMMUNITY PARTNERSHIPS

Community partnerships can nurture bidirectional relationships between community members and local health professionals and identify specific community needs and priorities. Community engagement in public health and social service initiatives has been successful at various levels in health promotion and addressing disparities.^7^ The involvement of communities in interventions focused on reducing health inequity enhances the design, delivery, and uptake by incorporating the needs, values, and preferences of patients and communities. Community partnerships may effectively encourage communities and patients to see a valuable role in diagnostic safety efforts by enhancing health care organizations’ accountability through community vigilance. While data on community engagement in patient safety are lacking, when clinicians know their activities are closely monitored by the communities they serve, they tend toward showing accountability and responsiveness to improve quality and safety.^8^ In academic medicine, there is an increasing focus on community engagement as part of the health care mission; however, few institutions have developed robust or transparent processes for authentic and sustained community partnership outside of research.

Community partnership in diagnostic safety efforts is facilitated by identifying active community champions. Community health workers (CHWs) serve as frontline public health workers, and trusted community members can enable access to healthcare systems and clinicians; strengthen team support by acting as a link between healthcare systems and communities; provide culturally appropriate health education information; and advocate for the healthcare needs of people in underserved communities. CHWs can be valuable resources in reducing diagnostic inequity by building bridges between health systems and communities and help to improve the relevance, acceptability, and accessibility of health services. For instance, CHWs can assist patients experiencing delays in diagnostic evaluation, improve appointment attendance, address language barriers, and reinforce the importance of referrals, testing, and follow-up. CHWs can improve health outcomes and address social determinants of health (SDOH) when adequately integrated into clinical care teams.^9^ Future work should explore how CHWs can be better integrated into the diagnostic team to help fill gaps in the diagnostic process.

## CONNECTED CARE

Using telehealth to engage patients in the diagnostic process may eliminate traditional barriers to care (e.g., accessibility, transportation challenges). Although telehealth has helped meet patients’ care expectations, pre-COVID19 adoption constraints and disparities have remained, such as provider concerns, payment issues, patient access and digital literacy, and other structural and organizational barriers. Studies of telehealth associations with race and rurality have had mixed results,^10^ and rural patients who use telehealth visits are more likely to be young, white, and have insurance. Patients without exposure to telehealth have concerns regarding the quality of the encounter and whether diagnoses can be made virtually.^11^ Safer “ telediagnosis” thus requires additional considerations.^12^

Despite the rapid telehealth implementation during the pandemic, limited local-level exploration of disparities in access to the necessary technology will limit diverse patient populations’ participation. Disparities in access to the required technology, ability to use the technology, and telehealth literacy threaten to worsen health disparities. Since the pandemic began, ongoing initiatives focused on healthcare disparities, such as the role of SDOH, have received growing attention and are now more likely to be considered in decision-making around telehealth moving forward. Nevertheless, telehealth, through its multiple modalities, has promise for improving diagnostic equity. For instance, offering patients tools to support telehealth access (e.g., Wi-Fi access points) and developing reimbursement models that support providing increased telehealth access can help to reduce disparities by offering patients multiple entry points into the diagnostic process.^13^

In conclusion, diagnosis cannot be safe without equity. Given the prevalence of diagnostic errors and growing attention to longstanding issues of inequity in healthcare, a multipronged approach to address diagnostic equity is necessary. A strategy that includes fostering clinician advocacy for patients and themselves, trustworthy community partnerships, and accessible, multimodal connected care holds potential for identifying and addressing inequity in the diagnostic process.
